# A single oral dose of citalopram increases interoceptive insight in healthy volunteers

**DOI:** 10.1007/s00213-022-06115-7

**Published:** 2022-03-24

**Authors:** James J. A. Livermore, Clare L. Holmes, Gyorgy Moga, Kristian Adamatzky, Hugo D. Critchley, Sarah N. Garfinkel, Daniel Campbell-Meiklejohn

**Affiliations:** 1 School of Psychology, University of Sussex, Pevensey Building, Falmer, Brighton, BN1 9QH UK; 2 Donders Institute for Brain, Cognition and Behaviour, Radboud University, Nijmegen, the Netherlands; 3 Department of Neuroscience, Brighton and Sussex Medical School (BSMS), Brighton, UK; 4 Sackler Centre for Consciousness Science, University of Sussex, Brighton, UK; 5 Institute for Cognitive Neuroscience, University College London, London, UK

**Keywords:** Serotonin, Citalopram, Interoception, Metacognition, SSRI

## Abstract

**Rationale:**

Interoception is the signalling, perception, and interpretation of internal physiological states. Many mental disorders associated with changes of interoception, including depressive and anxiety disorders, are treated with selective serotonin reuptake inhibitors (SSRIs). However, the causative link between SSRIs and interoception is not yet clear.

**Objectives:**

To ascertain the causal effect of acute changes of serotonin levels on cardiac interoception.

**Methods:**

Using a within-participant placebo-controlled design, forty-seven healthy human volunteers (31 female, 16 male) were tested *on* and *off* a 20 mg oral dose of the commonly prescribed SSRI, citalopram. Participants made judgements on the synchrony between their heartbeat and auditory tones and then expressed confidence in each judgement. We measured three types of interoceptive cognition.

**Results:**

Citalopram increased cardiac interoceptive insight, measured as correspondence of self-reported confidence to the likelihood that interoceptive judgements were actually correct. This effect was driven by enhanced confidence for correct interoceptive judgements and was independent of measured cardiac and reported subjective effects of the drug.

**Conclusions:**

An acute change of serotonin levels can increase insight into the reliability of inferences made from cardiac interoceptive sensations.

**Supplementary Information:**

The online version contains supplementary material available at 10.1007/s00213-022-06115-7.

## Introduction

Interoception is the signalling, perceiving, and interpreting of internal physiological states. It is the afferent arm of allostasis, through which health and vitality are maintained by adaptations in physiology, cognition, and behaviour. Because interoception carries information about the state and needs of the body, it can influence how one ‘feels’, what one will choose, how one will react, what one learns, one’s sense of threat, and beliefs about the current state of the ‘self’ (Cameron 2002; Craig 2002; Seth 2013). The last decade has brought a renaissance of interoception research including its measurement, its neural substrates, and its influence (Garfinkel et al. 2015; Brener and Ring 2016; Schulz 2016; Khalsa et al. 2018; Allen and Tsakiris 2018). Little is known, however, about its psychopharmacology.

Interoceptive changes are a transdiagnostic feature across multiple psychiatric conditions, including depressive, anxiety, and eating disorders (Ehlers 1993; Avery et al. 2014; Herbert and Pollatos 2019; Paulus et al. 2019; Eggart et al. 2019), a majority of which are treated to variable extent and degree of success, with selective serotonin reuptake inhibitors (SSRIs). The relationship between SSRIs and interoception is not well understood, but could be illuminated through the use of recent advances in interoception measurement and by observation of effects of different durations of treatment. In this experimental medicine study, we investigated the effect of an acute exogenous manipulation of serotonin levels on components of cardiac interoception. Through study of the link between SSRIs and cardiac interoceptive ability, we sought to provide the foundation for future research on the role of interoception in models of SSRI action. By conducting the study with an acute dose, we provide clues of the early effects of SSRI treatment. Through the study of healthy individuals, we establish these effects without confounds associated with disorders or other medications.

There are reasons to expect a relationship between serotonin and interoception. The serotonin system is strongly linked to homeostasis-regulating processes of digestion, temperature, respiration, bladder control, and stress (Berger et al. 2009), and is implicated in a range of cognitive and behavioural control processes (Deakin and Graeff 1991; Dayan and Huys 2009; Cools et al. 2011). Serotonergic neurons have demonstrated roles of signalling both reward and punishment (Cohen et al. 2015), and serotonin is thought to have regulatory effects on the influence of other systems on cognition and behaviour (Spoont 1992; Dayan and Huys 2009). Through interoception, bodily homeostatic states are communicated to the brain; this influences a wide variety of other cognitive processes (including the experience of both reward and punishment), and is putatively susceptible to the regulatory orchestration of other systems (such as by serotonin) (Craig 2002; Owens et al. 2018). Anatomically, the broad distribution of the serotonin system reflects its influence on other processes, and serotonin nuclei in the brainstem are well positioned to regulate communication between brain and body. Serotonergic antidepressants have been shown to modulate the experience of pain (Micó et al. 2006; Jann and Slade 2007), a domain which shows overlapping neural pathways with other interoceptive modalities (Craig 2002). In the other direction, visceral states may alter central serotonin availability, potentially by peripheral regulation of tryptophan metabolism (O’Mahony et al. 2015). There are also existing hints of a relationship between serotonin and cardiac interoception. In healthy individuals, a reduced correlation between neural and cardiac response to surprise, for instance, occurs when the serotonin precursor, tryptophan, is depleted (Mueller et al. 2012). Moreover, patients with social anxiety have reduced 5-HT_1A_ binding and increased serotonin availability in the insula cortex and the amygdala (Lanzenberger et al. 2007; Frick et al. 2015), key areas for interoception (Craig 2002; Schulz 2016) and cardiac influence on threat perception (Garfinkel et al. 2014), respectively. In panic disorder, which is related to metacognitive beliefs about one’s interoceptive sensitivity and internal sensations (Yoris et al. 2015), 5HT_1A_ binding is also decreased in the amygdala (Nash et al. 2008).

 SSRIs quickly change the synaptic availability of serotonin but can take weeks to bring about changes of mood, if this occurs at all (Cipriani et al. 2018). Studies of acute, single dose, effects of SSRIs provide insight into their effects during early stages of treatment. These studies, in healthy and clinical populations, provide for neurocognitive theories that could help early prediction of clinical outcomes from SSRI treatments (Kingslake et al. 2017). Current theories posit that early changes of perception, learning, memory and/or other cognition gradually reshape expectations and elevate mood (Skandali et al. 2018; Michely et al. 2020; Godlewska and Harmer 2021). These early effects might be underpinned by effects on interoception. Both interoception (Werner et al. 2009; Pfeifer et al. 2017; Critchley and Garfinkel 2017; Rae et al. 2018), and acute serotonin changes are associated with variabilty of learning, response inhibition, social perception, and decision-making (Chamberlain et al. 2006; Crockett et al. 2010; Scholl et al. 2017; Skandali et al. 2018; Godlewska and Harmer 2021). Twenty-one days of SSRI treatment reduces activation in interoception-related regions of the brain during the anticipation of aversive images (Simmons et al. 2009). Moreover, compared to unmedicated patients, patients with depression that are chronically medicated with SSRIs report greater intensity of cardiac interoceptive sensations while attending to them, than unmedicated patients (Burrows et al. 2022). The acute effects of SSRIs on distinct components of interoceptive processing, free from potential confounds related to a disorder, however, have not yet been described.

We therefore tested whether an SSRI could alter processing of interoceptive signals in healthy participants. Using a within-subject design, we tested the mechanistic link between acute modulation of serotonin levels on cardiac interoception. We studied participants on and off the SSRI, citalopram. Citalopram was chosen for its specificity to serotonin (3800 times the affinity to the norepinephrine transporter and 10,000 times the affinity to the dopamine transporter (Owens et al. 2001)), tolerability, and common use. SSRIs can, to variable degrees, ameliorate depressive symptoms (Cipriani et al. 2018), which are associated with blunted interoceptive processing (Pollatos et al. 2009; Avery et al. 2014). In the short term, single doses of SSRIs can also be associated with enhanced symptoms of anxiety (Grillon et al. 2007; Browning et al. 2007; Harmer et al. 2009), which is also associated with interoceptive enhancement (Dunn et al. 2010). As a result, we predicted that interoceptive abilities would increase with a single dose of an SSRI.

The heart has rich bidirectional connections with the brain and provides an easily measurable physiological readout with high temporal resolution. As a result, cardiac interoception is commonly tested (Tsakiris 2017). Since cardiac interoception could also have a relationship with serotonin (Mueller et al. 2012) and was the interoceptive domain available to our expertise and facilities, it was tested in the present study. Interoceptive ability was quantified using a heartbeat discrimination task. This requires a participant to attend to their heart and report whether auditory tones are in or out of time with their heartbeat, and then self-rate confidence in that judgement. A recent advance for interoception research was the recognition that different dimensions of interoception can vary independently in human experiments and provide discriminating markers for pharmacological testing (Garfinkel et al. 2015; Khalsa et al. 2018). We focus on three: interoceptive accuracy (correct judgements about internal sensations), confidence in the judgements, and interoceptive insight (metacognitive evaluation of interoceptive accuracy i.e., correspondence of confidence to judgement accuracy). High interoceptive insight can result from high confidence when judgements that are based on interoception are accurate and low confidence when judgements are inaccurate. Processes represented by interoceptive insight have the potential to be the basis of top-down regulation of interoception on cognition and behaviour (Seth 2013; Owens et al. 2018; Allen and Tsakiris 2018).

## Methods

### Experimental design

This study used a double-blind, placebo-controlled, within-subject, cross-over design. Participants underwent two test sessions, at least 1 week apart, under medical supervision. In one session, they ingested 20 mg citalopram (Cipramil) in a cellulose capsule, with extra space filled with microcrystalline cellulose which is an inactive ingredient of the citalopram tablet. In the other session, they received placebo (an identical capsule containing only microcrystalline cellulose). No-one who had contact with participants was aware of the treatment order, which was pseudo-randomized, balanced for sex, and coded by a researcher who was not present during testing. Capsules were manufactured according to good manufacturing practice (European Medicines Agency 2018).

### Participants

On a separate occasion, prior to testing, prospective participants undertook a screening session with a health questionnaire, heart rate and blood pressure monitoring by a medical doctor, and a structured clinical interview by a lead researcher (JL) to screen for any undiagnosed psychiatric conditions (Mini International Neuropsychiatric Interview; MINI (Sheehan et al. 1998)).

Exclusion criteria included: age under 18 or over 35 years; history of mental disorder (including anxiety disorder, depression, eating disorder, psychosis and substance abuse disorder); presence of significant ongoing medical condition (including migraine, diabetes, epilepsy, glaucoma and hypertension); pregnancy or breastfeeding; currently taking any medication (excluding oral contraceptive pill); first-degree family history of bipolar disorder; MINI indication of: major depressive episode, manic episode, panic disorder, social phobia, obsessive compulsive disorder, posttraumatic stress disorder, alcohol dependence, substance dependence, mood disorder with psychotic features, psychotic disorder, anorexia nervosa, bulimia nervosa, generalized anxiety disorder, or antisocial personality disorder. Participants were instructed to abstain from alcohol or caffeine in the preceding 12 h before the start of all test sessions.

Fifty-one participants were recruited. Each participant was asked if they could feel their own pulse in their finger with the apparatus in place. Three participants were excluded for feeling their pulse in their finger against the apparatus and one for technical errors preventing data collection. Forty-seven participants were successfully tested twice (mean age 23 ($$SD=3.9)$$, 31 females, mean weight 64 kg ($$SD=10.9$$). Given the weights of recruited participants, the average citalopram dose was 0.34 mg/kg ($$SD=.05$$). Testing was conducted in two separate locations (test cubicles) and no difference of result was observed between locations. This study received ethical approval from the University of Sussex Sciences & Technology Cross-Schools Research Ethics Committee (ER/JL332/3, ER/JL332/9). Participants gave informed written consent.

### Procedure

Cardiac interoception was measured as part of a battery of tasks, including information sampling, visual metacognition (see supplementary information), and social decision-making. Behavioural testing was timed to begin at 3 h after administration, corresponding to estimated peak plasma levels (Milne and Goa 1991).

Citalopram can exhibit side effects (typically mild at 20 mg) including nausea, headache, and dizziness (Ekselius et al. 1997). Visual analogue scales (VAS; from 0 to 100) assessed the presence of these three somatic effects (nausea, headache, dizziness). Additionally, five emotion/arousal related effects were assessed with VAS scales between pairs of antonyms: alert − drowsy, stimulated − sedated, restless − peaceful, irritable − good-humoured, anxious − calm. Each measure was recorded three times: immediately following dosing, at the start behavioural testing and at the end. Mean scores for the two testing times were used in analyses, with paired t-tests to analyse whether significant differences occurred between citalopram and placebo sessions. Cardiac measures of heart rate (HR) and heart rate variability (the standard deviation of HR across intervals) were calculated at baseline and test time. Citalopram has been reliably shown to not affect blood pressure without interaction with other drugs (Watts et al. 2012; Zhong et al. 2017) so blood pressure was not a dependent measure.

### Task

Participants were connected to a fingertip pulse oximeter to monitor cardiac events (Xpod with 8000SM sensor, Nonin Medical Inc., Minnesota, USA). We used an interoception task used previously and programmed within MATLAB (version 2018a, MathWorks) (Hart et al. 2013).

The interoception task (Katkin et al. 1982; Garfinkel et al. 2015) is a two-alternative forced choice task, often called the heartbeat discrimination task. Participants were instructed beforehand that the computer would play a set of tones that would be in or out of sync with their heartbeat. During each trial, their heartbeat was measured in real-time, while a computer played a set of ten tones at either 250 ms or 550 ms after the R-wave (Payne et al. 2006). These timings correspond respectively to judgements of maximum and minimum simultaneity (i.e., synchronous or delayed) between stimulus presentation and heartbeat (Wiens and Palmer 2001) (see discussion for consideration of variable interval approaches). Following each trial, the participant was directed to respond to whether the tones were in or out of time with their heartbeats, and how confident they were in that answer using a VAS scale ranging from ‘total guess’ to ‘complete confidence’ on a scale of 1 to 10. Synchronous and delayed trials were presented in a pseudorandomized order. Twenty trials (10 synchronous and 10 delayed) were completed in each session. Performance on 20 trials correlates at $r=.7$ with performance on 100 trials (Kleckner et al. 2015) or at $r=.85$ with performance on 50 (Jones et al. 1987).

### Analysis

Accuracy scores were calculated by taking the mean number of correct responses for the session and dividing by the number of trials, resulting in a proportion correct. Confidence scores were computed as the mean of the trial-wise confidence VAS measure. Interoceptive insight was calculated by comparing confidence in correct choices and confidence in incorrect choices between drug and placebo conditions. The effects on correct and incorrect response confidence can be collectively summarized as the area under the receiver-operating characteristic curve (AUC, MATLAB v R2020a), measuring the ability of confidence discriminate correct and inaccurate responses, independent from and unbiased by individual differences of confidence. Note AUC curves of accuracy (choice predicting correctness) provide the same information as proportion correct and so the more intuitive proportion correct was used to report accuracy. For further investigation of interoceptive insight, we independently analyzed the effects of citalopram on confidence in correct judgements and confidence in incorrect judgements.

We analyzed within-subject differences between drug and placebo with repeated measures ANOVAs (JASP v 0.14). Potential confounding variables, including gender, treatment order, VAS scales and HR/HRV, were examined for significant main effects between drug conditions. Where effects were found at or near the significance threshold, we conducted ANCOVAs to determine whether they exerted influence on outcome measures. Further testing of their influence was then conducted by mediation analysis (see supplementary information).

## Results

Baseline performances on both tasks were consistent with previous studies (Table 1) (Garfinkel et al. 2015). Citalopram reduced heart rate (beats per minute (bpm): $t\left(46\right)=3.9,p= .01$, mean diff $\Delta M=4.0)$, increased nausea (on 100-point scale: $t\left(46\right)=2.9, p=.01, \Delta M=4.7$) compared with placebo, and on trend increased anxiety (on VAS scale of calm to anxious (scale 0–100), $t\left(46\right)=-1.8, p=.09, \Delta M=4.3$. These variables were therefore included as covariates in a second analysis. Citalopram did not change heart rate variability or any other measured physiological or subjective state (see Table S1).

**Table 1:** Effects of citalopram on interoception task performance

	Mean (SD)					With change of HR, nausea, and anxiety covariates	
	Placebo	Citalopram	*F(1,46)*	*p*	${\eta }^{2}$	*F(1,42)*	*p*
Interoceptive accuracy	0.56 (0.13)	0.58 (0.16)	0.57	.45	.01	0.10	.75
Confidence	5.03 (1.85)	5.47 (1.48)	3.60	.06	.07	3.05	.09
Interoceptive insight	0.50 (0.18)	0.58 (0.17)	6.51	.01*	.12	5.09	.03*

^*^ p < 0.05

**Fig. 1:**
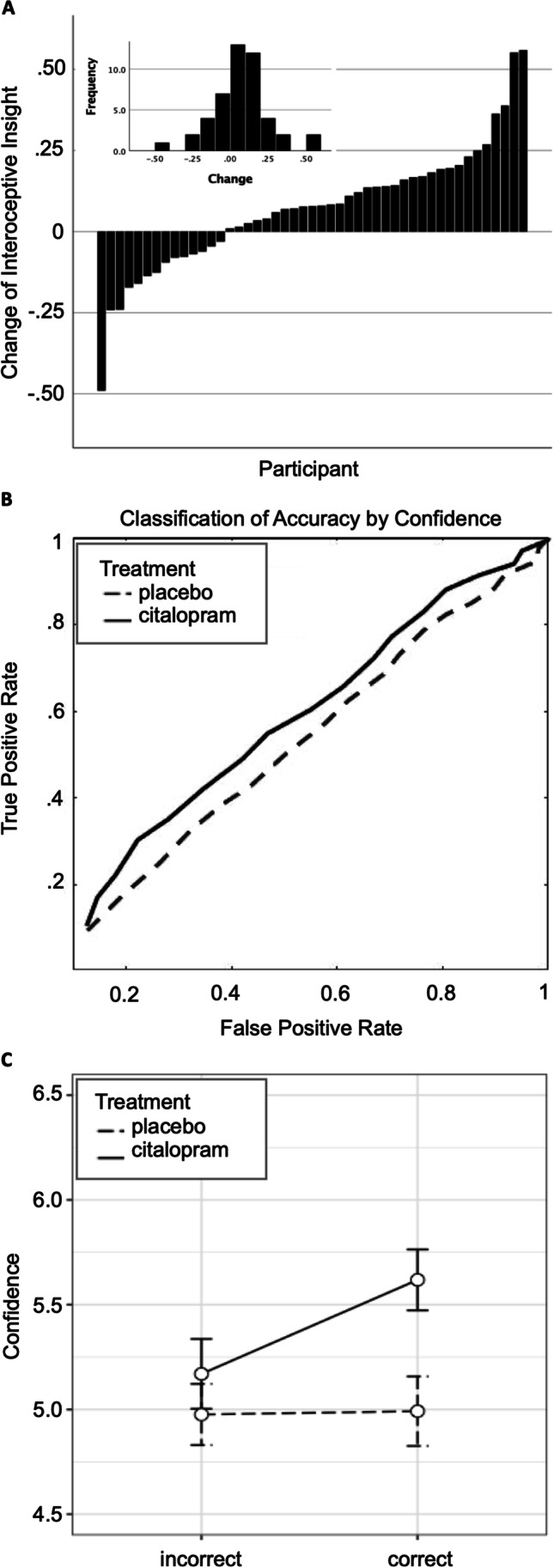
Effect of citalopram on interoceptive insight. **A** Change of interoceptive insight by each participant (citalopram - placebo). **B** Receiver operating characteristic curve representing confidence classification of interoceptive accuracy. **C** Confidence for correct and incorrect judgements on scale from 0 to 10. Error bars are within-subject standard error

Interoceptive accuracy was above 50% in both conditions (placebo $t\left(46\right)=3.2$, $p=.002$, citalopram $t\left(46\right)=3.6$, $p=.001$) but unchanged by citalopram (Table 1). Citalopram increased interoceptive insight with and without covariates of change to heartrate, nausea, and anxiety (Table 1, Fig. 1, Fig. S1). Approximately two-thirds of individuals showed this effect, with a medium-large effect size (${\eta }^{2}=.12)$ (Cohen 1988) (Fig. 1A). Changes of interoceptive insight appeared to be normally distributed (Fig. 1A). Inspection of Fig. 1C suggests that the effect on interoceptive insight was driven by increases of confidence for correct interoceptive judgements. There was an interaction between the effect of citalopram and whether the judgement was correct ($F\left(\mathrm{1,46}\right)=6.91$ $p=.01$; with covariates $F\left(\mathrm{1,42}\right)=3.55, p=0.07$). Confidence for correct judgements was higher on citalopram ($F\left(\mathrm{1,46}\right)=6.75, p =.01;$ with covariates $F\left(\mathrm{1,42}\right)=5.32, p =.02$) (Fig. 1C). Confidence for incorrect judgements did not significantly change ($ps$ > 0.25).

### Post-analysis

To better understand results, a number of further analyses were conducted. Visual inspection of participant data showed a small number of participants had greater absolute changes of interoceptive insight than the majority, both in the positive and negative direction (Fig. 1A). To satisfy curious readers, removing the three cases with the highest absolute values of change *increased* the statistical effect of citalopram on interoceptive insight described below. However, these cases were left in the final dataset because outlier test using the generalised extreme Studentised deviate procedure (Rosner 1983) at a threshold of 0.05 determined these values were not outlying.

There were no effects of gender (main *p* = 0.37, interaction p = 0.62) or treatment order (main *p* = 0.96, interaction *p* = 0.54).

There were no interaction effects between the effect of citalopram and (i) change of nausea (F(1,43 = 0.85, *p* = 0.36), (ii) heart rate (F(1,43 = 0.001, *p* = 0.98), or (iii) anxiety (F(1,43 = 0.07, *p* = 0.79) on interoceptive insight. The independence of interoceptive insight from subjective and physiological effects of the drug was confirmed by follow-up tests. We found near-zero correlation between interoceptive insight effects and changes of all physiological and subjective measures (Table S2), other than nausea which had a low non-significant correlation (*r*(45) = 0.14, *p* = 0.36). In restricted datasets with no difference between drug conditions on heart rate, anxiety, or nausea between drug conditions the effect of citalopram on interoceptive insight remained (see supplementary information). The effect of citalopram on interoceptive insight was not mediated by citalopram effects on accuracy, confidence, or any other measured effect of the drug (Fig. S2, Table S3).

## Discussion

A single dose of the SSRI citalopram enhanced awareness of the likelihood than an inference based on interoceptive information would be correct, demonstrating that an acute exogenous manipulation of serotonin levels is sufficient to change interoceptive insight. This relationship was independent of citalopram’s effects on heartrate or self-reported subjective states. Below, we consider potential mechanisms, future research directions, and methodological limitations.

We first examine how these effects may be implemented within existing models of interoception, with the caveat that this can only be speculative within the scope of the present study. The cognitive mechanism of citalopram’s effect on interoceptive insight will likely depend on the method or methods by which an individual determines their first-order interoceptive accuracy. For instance, if a particular allocation of attention (between sound, cardiac interoception, and other information) increases the likelihood of a correct choice, then improved retrospective awareness of attentional allocation (e.g. by citalopram) would improve insight into performance (Kanai et al. 2010). Knowing that one was paying attention on a trial should increase confidence that one was correct. Alternatively, a neurocomputational account is possible. The effects of citalopram may increase insight into qualities of the interoceptive information used to make the choice (Yeung and Summerfield 2012). Predictive coding models of interoception explain subjective feelings and allostasis as outputs of interactions between top-down predictions and bottom-up sensations from the body (Friston 2012; Seth 2013; Owens et al. 2018; Allen and Tsakiris 2018). A common theme of such models, is that top-down predictions can suppress or explain away signals encoded at lower levels. This leaves remaining signals, or ‘prediction errors’, to be broadcast forward hierarchically through layers of processing to adjust predictions and drive allostasis. The layers of processing are putatively implemented by similarly hierarchical neural networks. Importantly, the top-down predictions are also suggested to contain information about the expected precision (inverse variance) of the ascending prediction errors, which is integrally related to confidence in these signals (Kanai et al. 2015). That confidence is thought to be used to modulate their ‘gain’ which governs propagation up the hierarchy, under the principle that more precise information should be afforded a higher gain and thus exert a larger influence on resulting percepts (Feldman and Friston 2010; Moran et al. 2013; Kanai et al. 2015). Accordingly, increased confidence in one’s correct interoceptive choices due to citalopram may therefore reflect a more accurate readout (or prediction) of the lower-level precision of interoceptive information used to make the choice. If this enhanced confidence reflects a representation of ‘precision’ used to modulate gain of interoceptive prediction errors through the hierarchy, then it may also predict the flow of that interoceptive information. Serotonin is already thought to have regulatory effects on the flow of information in other systems (Spoont 1992; Dayan and Huys 2009). The precise pharmacological mechanism how such an effect would occur, however, is still not clear.

Importantly, due to the nature of the task, the effect of citalopram may have resulted from cognitive effects on processing *either* interoceptive information *or* interoceptive-exteroceptive signal integration.

The cardiac interoception task employed in this study is challenging. Accuracy was above chance in both treatment conditions, but only a minority performed above chance in both sessions. So, the effect of citalopram on interoceptive insight was observed in a condition of high uncertainty about first order performance. Therefore, citalopram’s effect could be primarily evident in conditions of high uncertainty about interoceptive sensations, which may account for a significant portion of interoceptive cognition, but this uncertainty could vary between individuals and contexts. This also provides a poor fit to signal detection theory approaches to metacognition analysis that assume that metacognitive insight arises from the same information and processes as the perceptual decision, necessitating above chance accuracy to interpret above chance metacognition (Barrett et al. 2013). Outside of that analytical framework, however, decision-making and metacognition can have different inferential goals and be represented by different anatomy (see Scott et al. (2014). They can also be receptive to distinct types of information or be determined by different processing of the same information. These differences can lead to situations of high metacognitive sensitivity despite low first-order accuracy (i.e. accuracy of the sensory decision) (Fleming and Daw 2017), and indeed, high metacognitive accuracy can been observed at robustly chance performance (a situation known as ‘blind insight’) (Scott et al. 2014). The effect of citalopram on interoceptive insight in this study therefore fits with specificity for a top-down effect, whereby citalopram alters blind (or near-blind) insight into interoceptive judgements, without improving the interoceptive judgements themselves. Greater confidence when one has been correct can help one decide when to use first order interoceptive judgements, but it does not provide all the tools required to make better judgements in the first place. The specificity of citalopram’s effect provides a possible pharmacological dissociation between first order judgements and metacognition that may be a new direction for the study of pharmacology in perception and consciousness.

One may ask whether citalopram’s effect is specific to interoceptive metacognition or if it is a general metacognitive effect. In a separate experiment, described in supplemental material, we did not observe an effect of citalopram in the same participants for exteroceptive visual metacognition (Table S4, Fig. S3). Correspondingly, the effect on interoceptive metacognition was larger than a metacognitive effect on the visual task. Reflecting this specificity, a significant correlation between subjects of metacognitive insight measures on the two tasks fell away on placebo falls away on citalopram. The design differences between the two tasks do not make them directly comparable (i.e., different numbers of trials and the requirement to compare perception in two different modalities in the interoception task (interoceptive/auditory)). Taken with caution, however, this may be the initial sign of the selectivity of serotonin’s effect on interoceptive insight rather than on metacognition in general.

### Future directions

Further research would be necessary to understand the neurological mechanism of this selective effect on enhanced interoceptive insight because its neural correlates are not yet known. A good place to look may be the relatively high cortical concentration of the serotonin transporter in insular regions of cortex (Beliveau et al. 2017), regions which are closely linked to attentiveness to interoceptive signals (Schulz et al. 2016) and show rapid increases in serotonin transporter binding following administration of citalopram (Lundberg et al. 2007). Recent work by Ray et al. (2021) has demonstrated that within the insula cortex, top down connections (anterior to posterior insula regions) are largely inhibitory while bottom up connections are largely excitatory. This pattern is enhanced with major depression severity (an effect corrected with behavioural therapy). In contrast, a similar pattern is depreciated with depression severity in exteroceptive networks. By affecting these balances, citalopram might influence representations of the precision and gain of ascending sensory information.

With respect to the pharmacology, at least two avenues merit further investigation. First, targeted blockade of serotonin transporters has indirect effects on other systems that could mediate effects on interoception (Zhou et al. 2005). Further studies are needed to investigate the roles of related neurotransmitters. Secondly, effects could stem from activation or inhibition of serotonin transmission. Acute SSRI administration can cause reductions or increases in serotonin transmission due to activation of 5-HT_1A_ auto-receptors, with variation of this effect across brain regions (Beyer and Cremers 2008). Further study is therefore also needed to understand effects of long-term treatment and different doses in which case the observed effect may persist, increase, decrease, or reverse.

By establishing a link between citalopram and interoception, the field has a new grounding for research exploring a common thread between seemingly distinct effects of serotonergic drugs, each of which has been independently linked to both interoception and serotonin, including: incentive processing (Marshall et al. 2019; Michely et al. 2020), decision-making (Werner et al. 2009; Crockett et al. 2010), response inhibition (Rae et al. 2018; Skandali et al. 2018), startle (Grillon et al. 2007; Schulz et al. 2016), and threat perception (Harmer and Cowen 2013; Garfinkel and Critchley 2016).

Our finding also provides a new grounding for future research within clinical disorders, with the potential to add interoception to existing neurocognitive models of SSRI clinical mechanisms of effect (Kingslake et al. 2017; Michely et al. 2020; Godlewska and Harmer 2021). It will be important to consider, however, that the translation of effects of an acute dose of citalopram in healthy adults to chronic doses in clinical populations will not be straightforward. Effects of chronic treatment with SSRIs on interoception in major depression disorder, for instance, likely depend on the interoceptive measure, symptoms, and comorbidity.

### Methodological considerations

Research published after this project was designed noted that higher reliability of interoceptive accuracy measures can be achieved with 40–60 trials, which provide accuracy scores that correlate exceptionally well with accuracy using 100 trials (r > 0.9). The accuracy on 20 trials in the present study is still reasonable (r > 0.7) (Jones et al. 1987; Kleckner et al. 2015). Importantly, the citalopram effect on interoceptive insight is not reliant on the reliable determination of a specific level of trait accuracy, but on trial-to-trial awareness of *when* accuracy was high. Effects on interoceptive accuracy itself could be present when more trials are used, but we would predict this is unlikely given the clear lack of effect. Secondly, this study assumed a common subjective timing of the sensation of *in sync* (at 250 ms interval from R wave) and *out of sync* (at 550 ms), based on previous research (Wiens and Palmer 2001). It is possible that individuals’ sensations of *in sync* beats varied from this norm and effects of citalopram on interoceptive accuracy were not picked up as accurately as they would be if more than two intervals were tested (Brener and Ring 2016). The within-subject design of this study mitigates this between-subject variance problem, with the assumption that timing of a subjective sense of in and out of sync beats is the same on and off citalopram. Some researchers may, however, prefer variable interval methods, that reduce variance and provide further insight into the precise nature of citalopram’s effects on interoceptive accuracy and awareness. Finally, this study focused on the heart. Further research is needed to show whether the serotonergic effect on cardiac interoception generalizes to other interoceptive domains.

## Conclusion

A single dose of citalopram can alter metacognitive insight into cardiac interoceptive processes.

## Supplementary Information

Below is the link to the electronic supplementary material.

Supplementary file1 (PDF 615 KB)

## Data Availability

All data, code, and materials used in the analysis are available any researcher for purposes of reproducing or extending the analysis. To download the data associated with this manuscript go to https://figshare.com/s/7154cc6d436e54154c37.

## References

[CR1] Allen M, Tsakiris M (2018). The Interoceptive Mind.

[CR2] Avery JA, Drevets WC, Moseman SE (2014). Major depressive disorder is associated with abnormal interoceptive activity and functional connectivity in the insula. Biol Psychiat.

[CR3] Barrett AB, Dienes Z, Seth AK (2013). Measures of metacognition on signal-detection theoretic models. Psychol Methods.

[CR4] Beliveau V, Ganz M, Feng L (2017). A high-resolution *in vivo* Atlas of the human brain’s serotonin system. J Neurosci.

[CR5] Berger M, Gray JA, Roth BL (2009). The expanded biology of serotonin. Annu Rev Med.

[CR6] Beyer CE, Cremers TIFH (2008). Do selective serotonin reuptake inhibitors acutely increase frontal cortex levels of serotonin?. Eur J Pharmacol.

[CR7] Brener J, Ring C (2016). Towards a psychophysics of interoceptive processes: the measurement of heartbeat detection. Philos Trans R Soc B: Biol Sci.

[CR8] Browning M, Reid C, Cowen PJ (2007). A single dose of citalopram increases fear recognition in healthy subjects. J Psychopharmacol.

[CR9] Burrows K, DeVille DC, Cosgrove KT (2022). Impact of serotonergic medication on interoception in major depressive disorder. Biol Psychol.

[CR10] Cameron OG (2002). Visceral Sensory Neuroscience.

[CR11] Chamberlain SR, Muller U, Blackwell AD (2006). Neurochemical modulation of response inhibition and probabilistic learning in humans. Science.

[CR12] Cipriani A, Furukawa TA, Salanti G (2018). Comparative efficacy and acceptability of 21 antidepressant drugs for the acute treatment of adults with major depressive disorder: a systematic review and network meta-analysis. Lancet.

[CR13] Cohen J (1988). Statistical Power Analysis for the Behavioral Sciences.

[CR14] Cohen JY, Amoroso MW, Uchida N (2015). Serotonergic neurons signal reward and punishment on multiple timescales. Elife.

[CR15] Cools R, Nakamura K, Daw ND (2011). Serotonin and dopamine: unifying affective, activational, and decision functions. Neuropsychopharmacology.

[CR16] Craig A (2002). How do you feel? Interoception: the sense of the physiological condition of the body. Nat Rev Neurosci.

[CR17] Critchley HD, Garfinkel SN (2017). Interoception and emotion. Curr Opin Psychol.

[CR18] Crockett MJ, Clark L, Hauser MD, Robbins TW (2010). Serotonin selectively influences moral judgment and behavior through effects on harm aversion. Proc Natl Acad Sci U S A.

[CR19] Dayan P, Huys QJM (2009). Serotonin in affective control. Annu Rev Neurosci.

[CR20] Deakin JF, Graeff FG (1991). 5-HT and mechanisms of defence. J Psychopharmacol (oxford).

[CR21] Dunn BD, Stefanovitch I, Evans D (2010). Can you feel the beat? Interoceptive awareness is an interactive function of anxiety- and depression-specific symptom dimensions. Behav Res Ther.

[CR22] Eggart M, Lange A, Binser MJ (2019). Major depressive disorder is associated with impaired interoceptive accuracy: a systematic review. Brain Sci.

[CR23] Ehlers A (1993). Interoception and panic disorder. Adv Behav Res Ther.

[CR24] Ekselius L, von Knorring L, Eberhard G (1997). A double-blind multicenter trial comparing sertraline and citalopram in patients with major depression treated in general practice. Int Clin Psychopharmacol.

[CR25] European Medicines Agency (2018) Good manufacturing practice. In: European Medicines Agency. https://www.ema.europa.eu/en/human-regulatory/research-development/compliance/good-manufacturing-practice. Accessed 8 Dec 2020

[CR26] Feldman H, Friston K (2010). Attention, uncertainty, and free-energy. Front Hum Neurosci.

[CR27] Fleming SM, Daw ND (2017). Self-evaluation of decision-making: a general Bayesian framework for metacognitive computation. Psychol Rev.

[CR28] Frick A, Åhs F, Engman J (2015). Serotonin synthesis and reuptake in social anxiety disorder: a positron emission tomography study. JAMA Psychiat.

[CR29] Friston K (2012). Predictive coding, precision and synchrony. Cogn Neurosci.

[CR30] Garfinkel SN, Critchley HD (2016). Threat and the body: how the heart supports fear processing. Trends Cogn Sci.

[CR31] Garfinkel SN, Minati L, Gray MA (2014). Fear from the heart: sensitivity to fear stimuli depends on individual heartbeats. J Neurosci.

[CR32] Garfinkel SN, Seth AK, Barrett AB (2015). Knowing your own heart: distinguishing interoceptive accuracy from interoceptive awareness. Biol Psychol.

[CR33] Godlewska BR, Harmer CJ (2021). Cognitive neuropsychological theory of antidepressant action: a modern-day approach to depression and its treatment. Psychopharmacology.

[CR34] Grillon C, Levenson J, Pine DS (2007). A single dose of the selective serotonin reuptake inhibitor citalopram exacerbates anxiety in humans: a fear-potentiated startle study. Neuropsychopharmacology.

[CR35] Harmer CJ, Cowen PJ (2013). ‘It’s the way that you look at it’—a cognitive neuropsychological account of SSRI action in depression. Philos Trans R Soc B: Biol Sci.

[CR36] Harmer CJ, O’Sullivan U, Favaron E (2009). Effect of acute antidepressant administration on negative affective bias in depressed patients. AJP.

[CR37] Hart N, McGowan J, Minati L, Critchley HD (2013). Emotional regulation and bodily sensation: interoceptive awareness is intact in borderline personality disorder. J Pers Disord.

[CR38] Herbert B, Pollatos O (2019). The relevance of interoception for eating behavior and eating disorders. The Interoceptive Mind.

[CR39] Jann MW, Slade JH (2007). Antidepressant agents for the treatment of chronic pain and depression. Pharmacotherapy.

[CR40] Jones GE, Jones KR, Rouse CH (1987). The effect of body position on the perception of cardiac sensations: an experiment and theoretical implications. Psychophysiology.

[CR41] Kanai R, Walsh V, Tseng C (2010). Subjective discriminability of invisibility: a framework for distinguishing perceptual and attentional failures of awareness. Conscious Cogn.

[CR42] Kanai R, Komura Y, Shipp S, Friston K (2015). Cerebral hierarchies: predictive processing, precision and the pulvinar. Philos Trans R Soc B: Biol Sci.

[CR43] Katkin ES, Morell MA, Goldband S (1982). Individual differences in heartbeat discrimination. Psychophysiology.

[CR44] Khalsa SS, Adolphs R, Cameron OG (2018). Interoception and mental health: a roadmap. Biol Psychiatry: Cogn Neurosci Neuroimaging.

[CR45] Kingslake J, Dias R, Dawson GR, et al (2017) The effects of using the PReDicT Test to guide the antidepressant treatment of depressed patients: study protocol for a randomised controlled trial. Trials 18. 10.1186/s13063-017-2247-2 10.1186/s13063-017-2247-2 PMC5701462 29169399

[CR46] Kleckner IR, Wormwood JB, Simmons WK (2015). Methodological recommendations for a heartbeat detection-based measure of interoceptive sensitivity. Psychophysiology.

[CR47] Lanzenberger RR, Mitterhauser M, Spindelegger C (2007). Reduced Serotonin-1A receptor binding in social anxiety disorder. Biol Psychiat.

[CR48] Lundberg J, Christophersen JS, Petersen KB (2007). PET measurement of serotonin transporter occupancy: a comparison of escitalopram and citalopram. Int J Neuropsychopharmacol.

[CR49] Marshall AC, Gentsch A, Blum A-L (2019). I feel what I do: relating interoceptive processes and reward-related behavior. Neuroimage.

[CR50] Michely J, Eldar E, Martin IM, Dolan RJ (2020). A mechanistic account of serotonin’s impact on mood. Nat Commun.

[CR51] Micó JA, Ardid D, Berrocoso E, Eschalier A (2006). Antidepressants and pain. Trends Pharmacol Sci.

[CR52] Milne RJ, Goa KL (1991) Citalopram. A review of its pharmacodynamic and pharmacokinetic properties, and therapeutic potential in depressive illness. Drugs 41:450–477 10.2165/00003495-199141030-00008 1711447

[CR53] Moran RJ, Campo P, Symmonds M (2013). Free energy, precision and learning: the role of cholinergic neuromodulation. J Neurosci.

[CR54] Mueller EM, Evers EA, Wacker J, Van Der Veen F (2012) Acute tryptophan depletion attenuates brain-heart coupling following external feedback. Front Hum Neurosci 6. 10.3389/fnhum.2012.00077 10.3389/fnhum.2012.00077 PMC3321412 22509162

[CR55] Nash JR, Sargent PA, Rabiner EA (2008). Serotonin 5-HT1A receptor binding in people with panic disorder: positron emission tomography study. Br J Psychiatry.

[CR56] O’Mahony SM, Clarke G, Borre YE (2015). Serotonin, tryptophan metabolism and the brain-gut-microbiome axis. Behav Brain Res.

[CR57] Owens MJ, Knight DL, Nemeroff CB (2001) Second-generation SSRIs: human monoamine transporter binding profile of escitalopram and R-fluoxetine. Biol Psychiatry 50:345–350 10.1016/s0006-3223(01)01145-3 11543737

[CR58] Owens AP, Allen M, Ondobaka S, Friston KJ (2018). Interoceptive inference: from computational neuroscience to clinic. Neurosci Biobehav Rev.

[CR59] Paulus MP, Feinstein JS, Khalsa SS (2019). An active inference approach to interoceptive psychopathology. Annu Rev Clin Psychol.

[CR60] Payne RA, Symeonides CN, Webb DJ, Maxwell SRJ (2006). Pulse transit time measured from the ECG: an unreliable marker of beat-to-beat blood pressure. J Appl Physiol.

[CR61] Pfeifer G, Garfinkel SN, Gould van Praag CD (2017). Feedback from the heart: emotional learning and memory is controlled by cardiac cycle, interoceptive accuracy and personality. Biol Psychol.

[CR62] Pollatos O, Traut-Mattausch E, Schandry R (2009). Differential effects of anxiety and depression on interoceptive accuracy. Depress Anxiety.

[CR63] Rae CL, Botan VE, Gould van Praag CD (2018). Response inhibition on the stop signal task improves during cardiac contraction. Sci Rep.

[CR64] Ray D, Bezmaternykh D, Mel’nikov M, et al (2021) Altered effective connectivity in sensorimotor cortices is a signature of severity and clinical course in depression. PNAS 118. 10.1073/pnas.2105730118 10.1073/pnas.2105730118 PMC8501855 34593640

[CR65] Rosner B (1983). Percentage points for a generalized ESD many-outlier procedure. Technometrics.

[CR66] Scholl J, Kolling N, Nelissen N, et al (2017) Beyond negative valence: 2-week administration of a serotonergic antidepressant enhances both reward and effort learning signals. PLoS Biol 15. 10.1371/journal.pbio.2000756 10.1371/journal.pbio.2000756 PMC5331946 28207733

[CR67] Schulz A, Matthey JH, Vögele C (2016). Cardiac modulation of startle is altered in depersonalization-/derealization disorder: evidence for impaired brainstem representation of baro-afferent neural traffic. Psychiatry Res.

[CR68] Schulz SM (2016) Neural correlates of heart-focused interoception: a functional magnetic resonance imaging meta-analysis. Philosophical Transactions of the Royal Society B: Biological Sciences 371:20160018. 10.1098/rstb.2016.0018 10.1098/rstb.2016.0018 PMC5062106 28080975

[CR69] Scott RB, Dienes Z, Barrett AB (2014). Blind insight: metacognitive discrimination despite chance task performance. Psychol Sci.

[CR70] Seth AK (2013). Interoceptive inference, emotion, and the embodied self. Trends Cogn Sci (regul Ed).

[CR71] Sheehan DV, Lecrubier Y, Sheehan KH, et al (1998) The Mini-International Neuropsychiatric Interview (M.I.N.I.): the development and validation of a structured diagnostic psychiatric interview for DSM-IV and ICD-10. The Journal of Clinical Psychiatry 59 Suppl 20:22–33;quiz 34–57 9881538

[CR72] Simmons AN, Arce E, Lovero KL (2009). Subchronic SSRI administration reduces insula response during affective anticipation in healthy volunteers. Int J Neuropsychopharmacol.

[CR73] Skandali N, Rowe JB, Voon V (2018). Dissociable effects of acute SSRI (escitalopram) on executive, learning and emotional functions in healthy humans. Neuropsychopharmacology.

[CR74] Spoont MR (1992). Modulatory role of serotonin in neural information processing: implications for human psychopathology. Psychol Bull.

[CR75] Tsakiris M (2017). The multisensory basis of the self: from body to identity to others. Q J Exp Psychol.

[CR76] Watts SW, Morrison SF, Davis RP, Barman SM (2012). Serotonin and blood pressure regulation. Pharmacol Rev.

[CR77] Werner NS, Jung K, Duschek S, Schandry R (2009). Enhanced cardiac perception is associated with benefits in decision-making. Psychophysiology.

[CR78] Wiens S, Palmer SN (2001). Quadratic trend analysis and heartbeat detection. Biol Psychol.

[CR79] Yeung N, Summerfield C (2012). Metacognition in human decision-making: confidence and error monitoring. Philos Trans R Soc B: Biol Sci.

[CR80] Yoris A, Esteves S, Couto B (2015). The roles of interoceptive sensitivity and metacognitive interoception in panic. Behav Brain Funct.

[CR81] Zhong Z, Wang L, Wen X (2017). A meta-analysis of effects of selective serotonin reuptake inhibitors on blood pressure in depression treatment: outcomes from placebo and serotonin and noradrenaline reuptake inhibitor controlled trials. Neuropsychiatr Dis Treat.

[CR82] Zhou F-M, Liang Y, Salas R (2005). Corelease of dopamine and serotonin from striatal dopamine terminals. Neuron.

