# Supplementary material for: A single oral dose of citalopram increases interoceptive insight in healthy volunteers
Source: Psychopharmacology (Berl). 2022 Mar 24;239(7):2289–98. doi: 10.1007/s00213-022-06115-7 (PMC9205807; doi:10.1007/s00213-022-06115-7)
Supplement: Supplementary file 1 — Supplementary file1 (PDF 615 KB) [file 213_2022_6115_MOESM1_ESM.pdf]

Supplementary Materials for:

A Single Oral Dose of Citalopram Increases Interoceptive Insight in Healthy Volunteers

Journal: *Psychopharmacology*

Authors:

James J A Livermore[1,2] , Clare L Holmes[1], Gyorgy Moga[1], Kristian Adamatzky[1], Hugo D Critchley [3,4], Sarah N Garfinkel [3,4,5], and Daniel Campbell-Meiklejohn[1]

Affiliations:

[1] School of Psychology, University of Sussex, Brighton, UK

[2] Donders Institute for Brain, Cognition and Behaviour, Radboud University, Nijmegen, Netherlands

[3] Department of Neuroscience, Brighton and Sussex Medical School (BSMS), Brighton, UK

[4] Sackler Centre for Consciousness Science, University of Sussex, Brighton, UK

[5] Institute for Cognitive Neuroscience, University College London, London, UK

Corresponding Author:
Daniel Campbell-Meiklejohn
School of Psychology
Pevensey Building
University of Sussex
Falmer
BN1 9QH
E: daniel.cm@sussex.ac.uk

Supplementary Results

Figs. S1 S2 S3

Tables S1 to S4

Supplementary Experiment

Supplementary Results

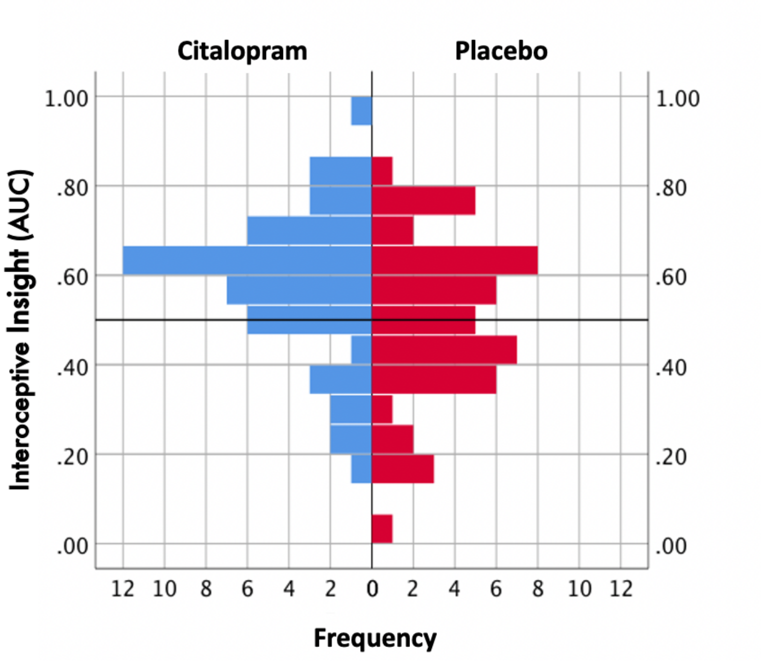

Fig S1. Distribution of Interoceptive Insight in both Conditions

<u>Somatic and psychological effects</u>

Table S1 shows the difference between scores in drug and placebo conditions on subjective ratings at test times.

Table S1: Mean VAS score (SD) at test time and contrasts between drug conditions. † full sample, ‡ restricted sample, * p < .05, ** p < .01

	**scale**	**placebo**	**citalopram**	***t*(46)**	***p***
**Full Sample**	nausea	4.47 (7.29)	9.2 (11.8)	-2.93	.005**
	headache	12.3 (17.3)	12.2 (17.6)	0.05	.96
	dizziness	9.4 (13.0)	11.0 (12.1)	-1.22	.23
	alert − drowsy	44.0 (18.2)	47.8 (19.5)	-1.27	.21
	stimulated − sedated	46.4 (15.6)	47.7 (14.9)	-0.54	.59
	restless − peaceful	64.3 (18.6)	61.2 (18.7)	1.02	.31
	irritable − good-humoured	63.9 (17.7)	64.8 (16.1)	-0.49	.62
	anxious − calm	71.9 (15.9)	67.6 (15.7)	1.75	.086

In addition to reporting results with change of heart rate, anxiety and nausea as covariates in the main analysis, we did further analysis to confirm the absence of influence of these factors on our findings. First, we tested for correlations of each change with the effect of citalopram on interoceptive insight (Table S2). No changes in cardiac or self-report variables between drug and placebo were significantly related to interoceptive insight. Next, we conducted a second analysis on restricted datasets, whereby cases were removed until a statistical comparison between citalopram and placebo exceeded p > 0.8. If data is restricted (N=31) to no difference of heart rate (F(1,30)=.01 , $p = .92, \Delta M - 0.08,$ SD 4.4 bpm reduction) between drug conditions, the effect of citalopram on interoceptive insight remains ($F(1,30) = 5.14$ $p = .03$). If data is restricted (N=37) to no difference on nausea (F(1,36)=.027, p = .87, ΔM = .13 SD 4.98), the citalopram effect on interoceptive insight remains ($F(1,36) = 5.0$, $p = .03$). If data is restricted (N=42) to no difference on anxiety (F(1,41) = .03, p =.87, ΔM = .33 SD 12.79), the effect of citalopram on interoceptive insight still remains (F(1,41) = 4.2, p =.046). As noted in the main text, analysis including all these factors simultaneously as covariates also preserves the interoceptive insight effect.

Table S2: Correlations between drug-placebo changes in cardiac/self-report variables and interoceptive insight

**Change of:**	**interoceptive insight**	
	***r*(45)**	**p**
heart rate	.01	.97
heart rate variability	-.01	.97
nausea	-.14	.36
headache	.00	.98
dizziness	.04	.82
alert−drowsy	.02	.90
stimulated – sedated	-.08	.59
restless – peaceful	.01	.95
irritable − good-humoured	-.01	.94
anxious – calm	.0	.88

Finally, we conducted a mediation analysis (Fig S2) to assess the possibility of indirect citalopram effects on interoceptive insight via other measured factors. We ran the mediation analyses using a within-subjects approach (MEMORE v2.1), which also includes both changes and average values of the mediator across conditions in the model (Montoya and Hayes 2017). This was conducted for all potential mediators showing differences between drug and placebo i.e., path B, or correlations between drug-placebo differences and task variables (path C). Results are reported in Table S3. No significant mediations were found.

Fig S2: Mediation analysis approach

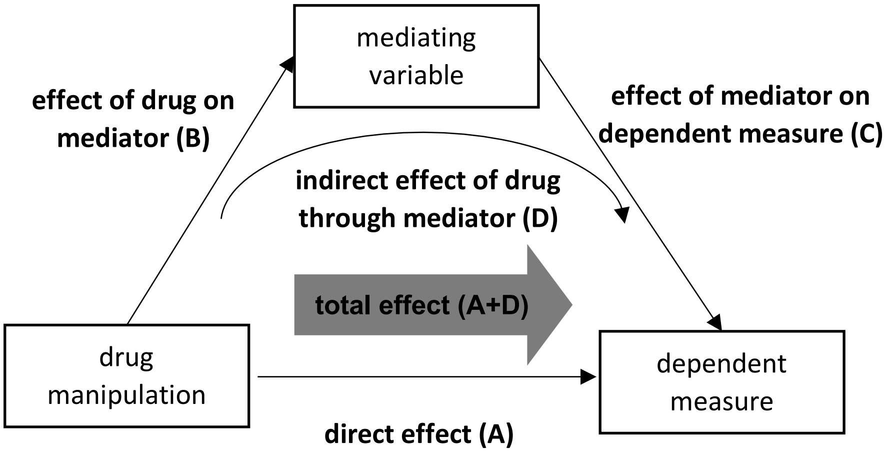

Table S3: mediation analyses on interoceptive insight. HR – heart rate, † in units of insight scores, ‡ in units of the mediator. If *b*ootstrap confidence intervals overlap zero, *indirect* effect is determined to be non- significant.

	Mediator(s)			
	**HR**	**nausea**	**anxiety**	**ALL 3**
direct citalopram effect on interoceptive insight *†*	0.08	0.07	0.07	0.08
t-stat	2.27	2.33	2.45	2.33
p	**.028**	**.024**	**.018**	**.024**
effect of citalopram on mediator *‡*	-3.99	4.71	4.29	
t-stat	-3.91	2.93	1.75	
p	**<.001**	**.005**	.086	
effect of mediator on interoceptive insight*†*	0.0013	<0.0001	.0002	
t-stat	0.27	0.01	.10	
p	.77	.99	.86	
indirect effect of citalopram on interoceptive insight through mediator (path D) *†*	-0.005	-0.0002	-.001	
Bootstrap Lower / Upper CI	-.05/.03	-.04/.02	-.01/.01	

Supplementary Experiment

The same participants performed a visual metacognitive Insight task (VMI) after the interoception task. The results are not directly comparable to the interoception task due to differences in task structure, but are included here because they indicate that effects of citalopram do not extend to visual metacognition. With caution, this demonstrates the potential for selectivity of citalopram effects on *interoceptive* metacognition.

Methods

VMI task: The visual task was taken from (Fleming et al. 2014). Participants were shown circles containing dots and instructed to indicate which contained more. Following each trial, they were asked to indicate their confidence in the previous response on a Likert scale. 200 trials were conducted in 8 blocks, with a self-timed rest every 25 trials. The difficulty was staircased over the course of the task, with the difference in numbers of dots (Δd), adjusted to target a mean rate of correct answers of 70%, to keep a consistent level of difficulty between participants. One randomly selected circle always held 50 dots. After two consecutive correct responses, Δd was decreased by one dot; after one incorrect response, Δd was increased by one dot.

We calculated Δd corresponding to the number of dots differing between the two circles necessary to maintain 70% accuracy as a measure of performance. Staircasing was successful: mean accuracy was .71 ($SD = .02$) on placebo and .72 ($SD = .02$) on citalopram. Confidence scores were recorded on each trial. Using performance and confidence ratings (1 to 6), we calculated visual metacognitive insight (VMI) as AUC, corresponding to the interoceptive insight measure. This common measure allowed a direct comparison of citalopram effects on cardiac interoception and visual exteroception. We could then look specifically for changes of confidence on correct and incorrect judgements.

We also calculated visual metacognitive sensitivity (meta d') and efficiency (meta d' / d') for reference to previous research (Green and Swets 1966; Fleming and Lau 2014). Correlation of VMI and meta d' in the placebo condition was $r = .92$, and correlation between VMI and meta d'/d' was $r = .86$. We used GLMs including order, nausea, heart rate and anxiety as regressors of no interest to model within-subject differences between drug and placebo. We also examined the effects on confidence independently in correct and incorrect choices.

For comparison of VMI with interoceptive insight controlling for the difference in accuracy and number of trials between tasks, we completed a supplementary analysis on VMI. 1000 random draws of 20 samples each were taken from each participant separately for drug and placebo sessions, weighted to include correct and incorrect trials at the same proportion as that person's interoception task accuracy, in the same session. Computations on insight for each sample were made and the average of these used in statistical comparison with interoceptive insight at the same level of accuracy.

To test if citalopram effects on interoception were over and above a general effect on metacognition, we performed a post hoc analysis of interoceptive insight with the addition of change of visual metacognition as a covariate in our original analyses.

<u>Results</u>

Interoceptive insight correlated with its equivalent measure in visual perception (VMI) ($r(45) = .35, p = .02$) in the placebo condition indicating some consistency between measures, but this relationship disappeared on citalopram ($p = .85$).

Given an observation of near significant interaction with order of treatment for some measures of visual metacognition, order was included as a between subject factor in all models in addition to change of nausea, heart rate and anxiety.

A comparison of citalopram effects between interoceptive insight and VMI demonstrated a significant drug x task interaction ($F(1,45) = 6.56, p = .014$), with a greater effect on interoception. There was no effect of citalopram on VMI (Table S4). This remains the case for subsets of visual perception data matching interoceptive task accuracy (20 visual task trials with same mean accuracy as interoceptive task performance).

Inclusion of visual metacognition changes in the original interoceptive insight analysis (in addition to anxiety, nausea and heart rate) as a covariate did not predict or change the effect of citalopram on interoceptive insight (F(1,42)=5.78, p = .02). Change of visual metacognition did not predict the citalopram effects on interoceptive insight (F1,42) = 0.22, p = .64). There was no interaction effect between effects of citalopram on VMI changes and interoceptive insight (F(1,42)=1.60, p = 0.21).

Table S4: Repeated measures ANOVA conditional main effect for all exteroception (visual) metacognition task. Full factorial models include order as a between subject factor, change of nausea, change of heartbeat, and change of anxiety, interactions of each covariate and factor with drug condition.

	placebo	citalopram	conditional main effect $F(1,45)$	p
**Δd**	5.49	5.74	0.00	.99
**confidence (1-10 scale)**	5.84	5.66	0.50	.48
**confidence correct choices**	6.19	5.99	0.91	.35
**confidence incorrect choices**	5.00	4.79	.01	.94
**VMI (AUC)**	0.65	0.65	.50	.49
**meta d'**	0.96	0.96	0.48	.49
**meta d' / d'**	0.79	0.77	1.19	.28

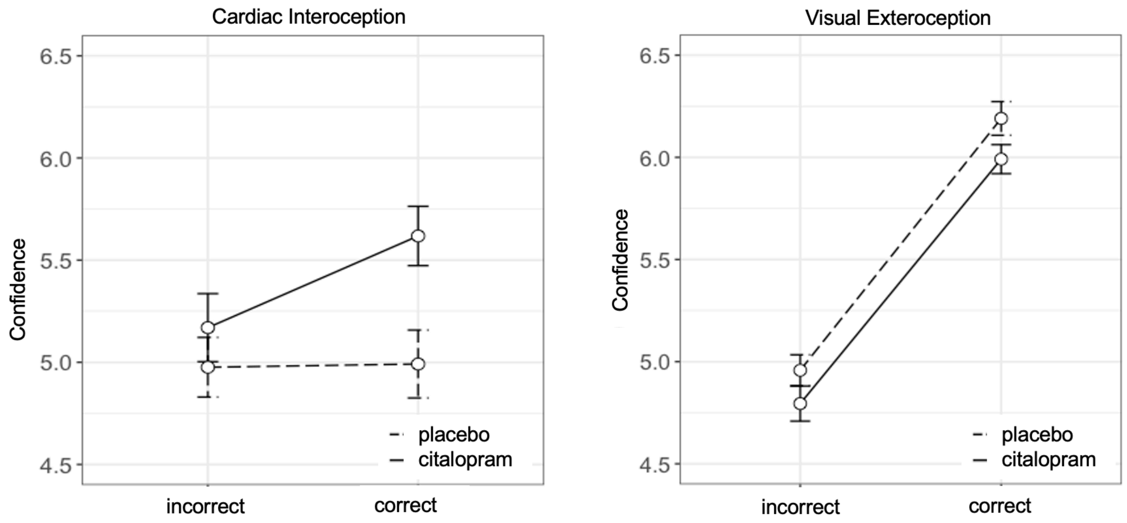

Fig S3. Confidence for correct and incorrect judgements for cardiac interoception (same as Fig 1, main paper) and visual exteroception, on scale from 0 to 10. Error bars are within-subject standard error.

Fleming SM, Lau HC (2014) How to measure metacognition. Front Hum Neurosci 8:. https://doi.org/10.3389/fnhum.2014.00443

Fleming SM, Ryu J, Golfinos JG, Blackmon KE (2014) Domain-specific impairment in metacognitive accuracy following anterior prefrontal lesions. Brain 137:2811–2822. https://doi.org/10.1093/brain/awu221

Green DM, Swets JA (1966) Signal detection theory and psychophysics. John Wiley, Oxford, England

Montoya AK, Hayes AF (2017) Two-condition within-participant statistical mediation analysis: A path-analytic framework. Psychol Methods 22:6–27. https://doi.org/10.1037/met0000086
